# Musculoskeletal modeling and humanoid control of robots based on human gait data

**DOI:** 10.7717/peerj-cs.657

**Published:** 2021-08-09

**Authors:** Jun Yu, Shuaishuai Zhang, Aihui Wang, Wei Li, Lulu Song

**Affiliations:** 1 Zhongyuan-Petersburg Aviation College, Zhongyuan University of Technology, Zhengzhou, China; 2 School of Electric and Information Engineering, Zhongyuan University of Technology, Zhengzhou, China

**Keywords:** Human-gait, Musculoskeletal model, OpenSim, Humanoid control, Lower limb rehabilitation exoskeleton, Motion capture

## Abstract

The emergence of exoskeleton rehabilitation training has brought good news to patients with limb dysfunction. Rehabilitation robots are used to assist patients with limb rehabilitation training and play an essential role in promoting the patient’s sports function with limb disease restoring to daily life. In order to improve the rehabilitation treatment, various studies based on human dynamics and motion mechanisms are still being conducted to create more effective rehabilitation training. In this paper, considering the human biological musculoskeletal dynamics model, a humanoid control of robots based on human gait data collected from normal human gait movements with OpenSim is investigated. First, the establishment of the musculoskeletal model in OpenSim, inverse kinematics, and inverse dynamics are introduced. Second, accurate human-like motion analysis on the three-dimensional motion data obtained in these processes is discussed. Finally, a classic PD control method combined with the characteristics of the human motion mechanism is proposed. The method takes the angle values calculated by the inverse kinematics of the musculoskeletal model as a benchmark, then uses MATLAB to verify the simulation of the lower extremity exoskeleton robot. The simulation results show that the flexibility and followability of the method improves the safety and effectiveness of the lower limb rehabilitation exoskeleton robot for rehabilitation training. The value of this paper is also to provide theoretical and data support for the anthropomorphic control of the rehabilitation exoskeleton robot in the future.

## Introduction

The main causes of limb motor dysfunction in patients include stroke and limb injury (Sousa et al., 2011). The sequelae of this injurious seriously affect the quality of life for patients and their families. Worldwide, there are a large number of patients with limb dysfunction caused by various accidents (Bai et al., 2019). Meanwhile, the current global population is facing a very serious aging situation (Fuster, 2017). The number of patients with limb dysfunction is further increasing with the aging society. The lower limbs play a crucial role in our lives. The lower limb training helps to expand the scope of daily activities of the recovered person and reduce the risk of falling. Therefore, in the rehabilitation treatment of the limbs, the rehabilitation treatment of lower limbs is especially essential. Studies have shown that patients with limb motor dysfunction will recover their normal walking ability after a certain amount of scientific training at an appropriate time after injury (Mekki et al., 2018; Chen et al., 2016; LI & JIANG, 2010). However, the rehabilitation training work for patients with limb dysfunction in the later period is heavy, the existing medical staff cannot complete this huge task. The emergence of rehabilitation exoskeleton provides an effective solution to solve these social problems (Bernhardt et al., 2017; Coleman et al., 2017), fills the gap in the number of medical staff, and brings hope to the majority of patients with limb dysfunction. At present, the exoskeleton robot for lower limb rehabilitation training mostly uses force/position control or trajectory planning method (Shi et al., 2019; Bernhardt et al., 2005). Although the existing exoskeleton training process is mostly mechanized rigid training, the complexity of the human movement process determines that the exoskeleton is difficult to track the human movement track in the training process. Therefore, the traditional exoskeleton rehabilitation training cannot effectively meet the needs of patients with lower limb motor dysfunction.

In order to solve this problem, current researchers have proposed a musculoskeletal model of human-based on the characteristics of movement mechanisms to improve the effectiveness of rehabilitation training. The human musculoskeletal system is a complex non-linear, multi-redundant system, which is difficult for non-human physiology researchers, then most lower limb rehabilitation exoskeleton robot researchers rarely analyze the real human gait and human musculoskeletal model. The open-source software OpenSim developed by Stanford University brings a feasible solution for non-human physiology researchers. Seth et al. (2018) have jointly developed OpenSim, which can create a musculoskeletal model and then predict new motions through the model and perform motion analysis. OpenSim is the software based on computational modeling and simulation of biomechanical systems (Seth et al., 2018). Based on OpenSim, Guo et al. (2020) studied the biomechanical characteristics of human lower limbs at different speeds and different weights, performed gait simulation, and proposed joint torque and muscle activation during walking (Saul et al., 2015). Space circulation characteristics and biomechanical characteristics are the main content of gait analysis research. Researchers employed OpenSim to perform musculoskeletal modeling and analyze the joint kinematics and muscle force characteristics of gait (Wang et al., 2018). Cardona & Cena (2019) studied and analyzed the biomechanics of the lower limbs of the human body, and estimated the kinematics and dynamics parameters of healthy gait and pathological gait. Zhou et al. (2020) combined the human musculoskeletal model and exoskeleton modeling control, then conducted simulation research on exoskeleton design and control methods with humans in the loop (Branson et al., 2010). Humphreys (2019) uses OpenSim to test in an environment lacking measurement test data and microgravity to generate predictive kinematics. It is of great significance to study the coupling and synergy between the exoskeleton and humans. Dembia et al., (2017) employed OpenSim to simulate auxiliary equipment and reduce the metabolic cost of weight-bearing walking through simulation; this research will provide help for experimenters in the manufacture of exoskeleton devices. In the field of human body mechanism analysis and research, OpenSim has been widely used. However, most of these studies and applications start from the software itself to simulate and analyze motion, the results of OpenSim simulation analysis are rarely used for extended applications in the field of rehabilitation exoskeleton.

**Figure 1:**
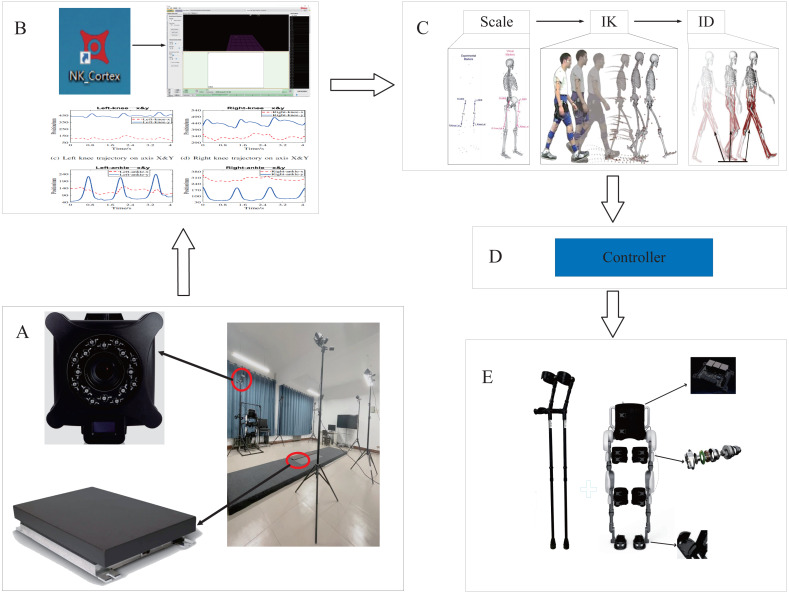
The schematic diagram of the principle.

Therefore, this paper expands the results of OpenSim simulation analysis and applies them to lower extremity rehabilitation exoskeleton robots. Starting from the human body motion mechanism, the human body kinematics analysis is carried out, and a PD control strategy based on real gait and musculoskeletal model is proposed. The schematic diagram of the principle is shown in Fig. 1. In this picture, A represents gait data acquisition, B represents data preprocessing, C represents modeling and analysis, D represents the controller, E represents the robot. First, this paper uses the NOKOV motion capture system and force measurement platform to collect normal human gait data, and preprocess the collected data. Then, the processed gait data was imported into OpenSim, and the musculoskeletal model of the experimental object was established for human kinematics and dynamic analysis, moreover obtained the mechanical characteristics of human motion. Finally, the human motion mechanical characteristics are proposed to control the torque of the lower limb exoskeleton robot based on the PD controller, and the error-free tracking is achieved by adjusting the controller parameters. This method improves the flexibility of the exoskeleton robot movement and meets the anthropomorphic design requirements of rehabilitation training.

The Ethics Committee of the School of Electronic Information, Zhongyuan University of Technology (approval batch number: ZUTSEI202008-001), approved this research protocol, and all participating patients signed an informed consent form.

### Analysis of the mechanism of human lower limb movement

#### Human gait data collection

At present, the motion capture system is divided into five categories according to the principle: mechanical motion capture system (Wu et al., 2005), acoustic motion capture system, electromagnetic motion capture system (Guo et al., 2011), inertial motion capture system (Kim & Nussbaum, 2013) and optical motion capture system (Kurihara et al., 2002; Guerra Filho, 2005; Kirk, O’Brien & Forsyth, 2005). Among them, the optical motion capture system is divided into two categories: motion capture system based on computer vision (optical non-calibration) and optical motion capture system based on mark point (optical calibration).

 This paper selected Nokov 3D infrared passive optical motion capture system with high accuracy and good effect after comparing several existing motion capture systems and combining with the needs for current research topic. In the scene set up by this system, the infrared camera is used to fully cover the experimental scene, infrared light is emitted by the infrared camera array in the process of data collection, and the position information of the reflective Maker points are captured in the experimental scene. In the process of collecting gait data, the experimental subject walks in the experimental scene with affixed Maker points. In order to meet the needs of the research, the experimental platform is equipped with a three-dimensional force measuring platform, which can synchronously collect the three-dimensional ground reaction force during the movement of the experimental object.

**Figure 2:**
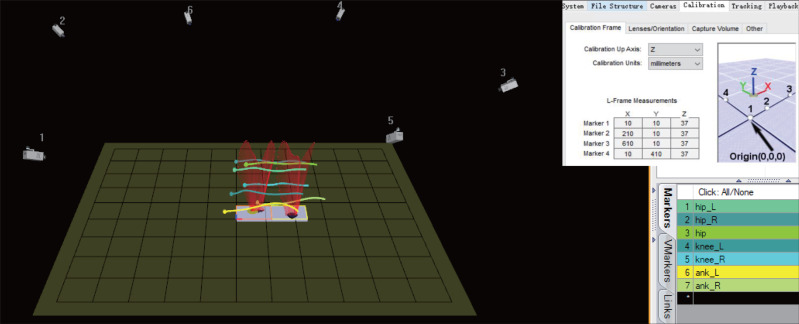
The human gait data collection scene.

Before collecting experimental data, the deployment of the experimental platform is also critical. The deployment of the camera position has a fatal impact on the experimental data (Kurihara et al., 2002). In the experiment, the influences of different camera arrangement methods on experimental data were tested. It was found that the best data is obtained by using the approximate circular camera arrangement. This arrangement allows each camera to maximize its utilization value. In the experiment, the cameras scene is arranged around the force measuring platform in an oval shape. The calibration origin is positioned as far as possible in the center of each camera’s field of view by adjusting the orientation of the cameras. The experimental collection scene is shown in Fig. 2.

#### Gait data processing

After the data collection is completed, it is preprocessed to ensure the completeness and accuracy of each frame of data. For missing data points, we had appropriate discarding or interpolation methods for processing. For severely missing data, the entire group will be deleted without applying.

**Figure 3:**
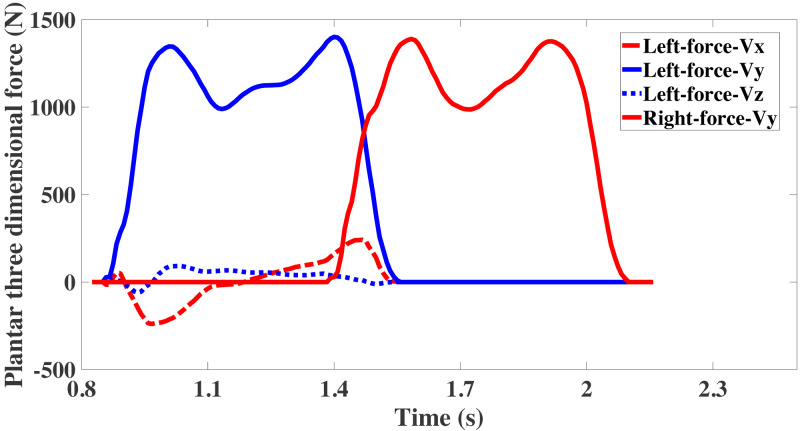
Component of plantar force on coronal, sagittal, and vertical axis.

The force was collected by three-dimensional measuring platform that is the force between foot and ground when the experimenter walks. During the process of gait collection, the force in the vertical direction is the most important force, it reflects the phenomenon of overweight and weightlessness during the gait cycle. The three-dimensional force as shown in Fig. 3. It can be found that the force between left foot and right foot with the ground is basically symmetrical on same axis. The data of the force platform is zero before the foot contacts it. Next, a small fluctuation is formed in the coronal and sagittal axes at first, then increases to a maximum and gradually decreases to zero. On the vertical axis, it increases rapidly, forming a bimodal curve similar to M, and then rapidly returns to zero too.

**Figure 4:**
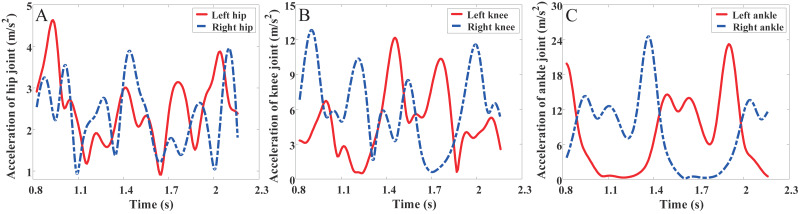
Combined acceleration of joints. (A) The acceleration of hip joint. (B) The acceleration of knee joint. (C) The acceleration of ankle joint.

The acceleration of the left and right joint has certain symmetry and periodicity, as shown in Fig. 4, it can be seen from the figure that the acceleration from the left leg joint and the right leg joint can basically coincide with each other in the case of 0.5 s of translation. During the walking process of the subject, the acceleration of hip joint points were maintained from 1 m/s^2^ to 5 m/s^2^. The acceleration of knee joint points were maintained from 0 m/s^2^ to 13 m/s^2^. The acceleration of ankle joint points were maintained from 0 m/s^2^ to 25 m/s^2^. It can be easily observed that the acceleration at the ankle joint points is greater than the acceleration at the knee joint points, and the acceleration at the knee joint points is greater than acceleration at the hip joint points. During walking, consistent with experience, the further away from the torso, the acceleration of the nodes greater. Here, a small idea is proposed: based on the acceleration of the joints, a body movement comfort function is designed to evaluate the patients’ comfort in the process of lower limb exoskeleton rehabilitation training. This will be a research direction in the next stage of this subject.

### Musculoskeletal modeling

In order to build musculoskeletal models and obtain relatively accurate biomechanical information, several common musculoskeletal modeling and computation software on the market, such as LIFEMOD (Huynh et al., 2015), OpenSim (Seth et al., 2011), Anybody (Damsgaard et al., 2006), SIMM software, were compared in the research process. The comparison results are shown in Table 1. It was found that OpenSim meets the needs of this study by comparison. It is an open sources free software developed by Stanford University. OpenSim calculates the motion process based on biomechanical knowledge and combining forward kinematics and inverse kinematics. OpenSim can be applied to human musculoskeletal model development, motion simulation, motion analysis, muscle strength calculation, normal and pathological gait analysis, etc.

**Table 1:** Musculoskeletal modeling software comparison.

Software	Main Features
LifeMOD	-Commercial: Yes.
	-Import Simulink from another: CAD, CATIA, Pro/E,
	SolidWorks, Unigraphics.
	-Inverse Kinematics utility.
	-Inverse Dynamics utility.
	-Allows simulations with implants.
OpenSim	-Commercial: NO (free).
	-Simulink Export: No native.
	-Muscle-driven forward dynamic (from data recorded).
	-Inverse Dynamics utility.
	-Inverse kinematic simulation.
	-Allows simulations with implants.
Anybody	-Commercial: Yes.
	-Simulink Export: No native.
	-Friction forces modeling.
	-Inverse Dynamics utility.
	-Allows simulations with implants.
SIMM	-Commercial: Yes.
	-Simulink Export: No native.
	-Real-time viewing.
	-Bone deformation modeling.
	-Inverse kinematics utility.

The first reason for using OpenSim modeling: rigid exoskeleton rehabilitation robot is a typical multi-input and multi-output complex mechanical system with nonlinear, strong coupling and other uncertain factors. There is a great inaccuracy when modeling the exoskeleton using a traditional linkage model. These inaccuracies are mainly reflected in the following aspects:

1. Mass moment;

2. Inertial matrix;

3. Changes in stiffness and damping (in the process of human–computer interaction);

4. Static friction force of the robot.

The second reason for using OpenSim modeling: compared with the exoskeleton robot, the musculoskeletal system of the human body is a more complex system with multiple redundancy, nonlinear and strong coupling. The most basic way of human movement is to pull the bones around the joints through muscle contraction to achieve the purpose of limb movement. Compared with the traditional connecting rod modeling, the musculoskeletal system modeling is more in line with the movement and texture characteristics of human body, and can better simulate some movements of human body, which is closer to the actual movement characteristics of human body. Using musculoskeletal model for simulation will get more accurate and reliable simulation results. Considering comprehensively, this paper chooses the musculoskeletal modeling method which is closer to the human body for data analysis and processing.

The model selected in this paper is based on the Gait 2354 model, which is from the OpenSim open-source community. This is a three-dimensional model with 23-degrees of freedom of the human musculoskeletal system. The model embodies the achievements of many predecessors. First, the original model is created by Thelen et al. The model uses Delp et al.’s definition of lower extremity joints (Delp et al., 1990), Anderson and Pandy’s low back joints and anthropometry (Anderson & Pandy, 1999; Anderson & Pandy, 2001), and Yamaguchi and Zajac’s plane knee model (Yamaguchi & Zajac, 1989a). The Gait2392 model features 92 muscle actuators to represent 76 muscles in the lower extremities and torso. For the Gait2354 model, the number of muscles was reduced by Anderson to improve simulation speed for demonstrations and educational purposes. Seth removed the patella to avoid kinematic constraints; insertions of the quadriceps are handled with moving points in the tibia frame.

#### Musculoskeletal model scaling

In this paper, the open-source musculoskeletal model was scaled to obtain the exclusive model equivalent to the experimental object. In order to ensure the accuracy of model scaling, the model was scaled several times. Finally, the accuracy of the model with a scaling error of less than one-thousandth is achieved.

**Figure 5:**
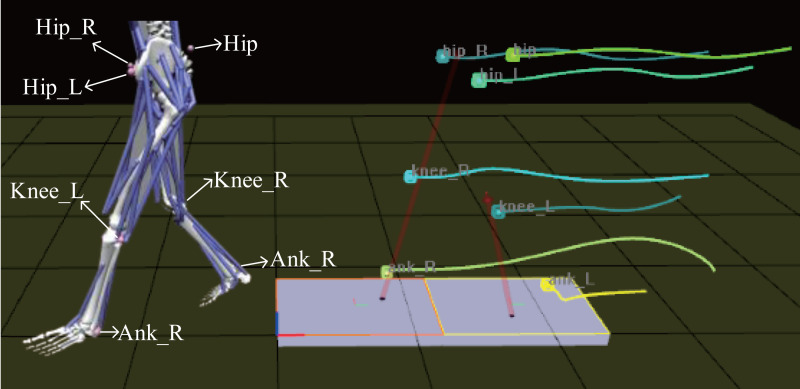
Markers.

Model scaling allows the open sources model to match our experimental subjects as closely as possible. In the scaling process, static data collected are mainly used in this paper (the experimental data collected while the experimental object is standing still). Before scaling the model, Maker points were added at the appropriate position on the model in accordance with the experimental object. As shown in Fig. 5. Meanwhile, these Maker points were connected to the model bones. In order to ensure the scaled model more accurate, the collected action data are processed in this paper. Through calculation, the left and right width of the pelvis and the length of the left thigh, left calf, right thigh and right calf were obtained(as shown in Table 2), where the mass and length are calculated through Zatsiorsky (Vaughan, Andrews & Hay, 1982) and Harless study (Drillis, Contini & Bluestein, 1964). Finally, the length of these body segments in the model was built.

All the above body segment lengths were obtained from the processing of experimental data, and the measured body segment lengths were averaged. The comparison shows that the data is relatively accurate. In this scaling process, we preserve mass distribution during scale, and change the scale weight of the makers to get a more accurate model.

#### Inverse Kinematics (IK)

Forward kinematics calculate the final position of the model by giving the initial position, velocity and acceleration of the model. The IK are opposite to the forward dynamics. IK figures out the motion process of the model based on the given position, including the change of physical quantity such as velocity and acceleration in this process.

**Table 2:** Subject’s physical information.

Body segment	Thigh(L)	Calf(L)	Thigh(R)	Calf(R)
Origin model segment mass (Kg)	9.3014	3.7075	9.3014	3.7075
Scaled model segment mass (Kg)	8.0441	3.2063	8.0441	3.2063
Body mass by Harless study (Kg)	7.67	2.925	7.67	2.925
Segment length by experiment (mm)	458.17	395.12	456.36	394.22
Length by Harless study (mm)	438.48	394.98	438.48	394.98

**Figure 6:**
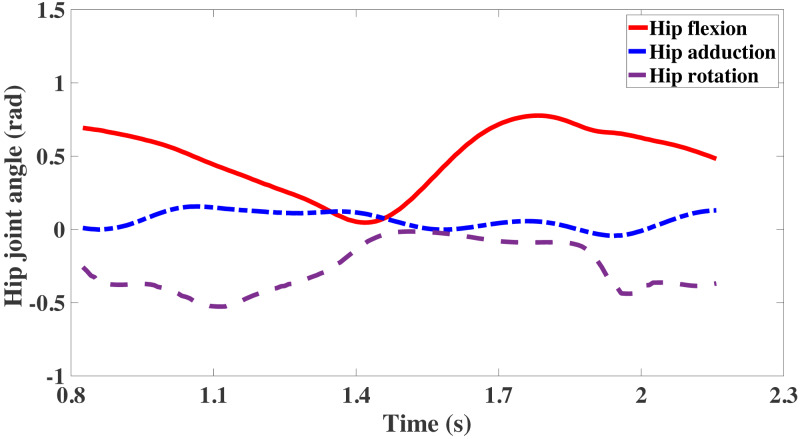
The adduction, flexion and rotation of hip joint.

The IK uses the motion capture data collected in the experiment (walk.trc). And the internal algorithm of the software was used for biological simulation, and the inverse solution was used to calculate the joint angle, pelvis tilt, et al. Among the lower limb movements of the human body, the hip joint movement is most complicated. The hip joint has three degrees of freedom. Therefore, the leg will perform three axial movements on the hip joint, including flexion and extension of the hip joint on the sagittal plane, adduction and abduction on the coronal plane, and internal rotation and external rotation direction of the thigh. During the gait cycle, the hip joint angles change as shown in Fig. 6. The hip joint of flexion is the movement in the sagittal plane, and its range of change is stable at 0 ∼ 0.8 rad. This movement change is the leading and effective movement of the hip joint during the gait, and the movement in this direction will drive the body to move forward. The hip joint of adduction changes smoothly in the gait, with only a slight fluctuation. The hip joint of rotation includes internal rotation and external rotation of the thigh during the gait. It can be seen that the data in this part has strong characteristics, and the range of variation is stable at −0.6 ∼ 0 rad. The hip joint of adduction and the hip joint of rotation have more personal characteristics related to personal habits and leg health conditions. It is also a key factor in judging whether the gait is abnormal. Through IK, the collected motion capture data can be matched with the calibration data of the experimental object, and the motion simulation process of the model can be optimized. The IK tool calculates universal coordinate values for each time step (frame) of the movement. Then, the model is positioned in the “best match” pose of the experimental marker time step. In other words, mark points in the collected motion process are matched with the motion capture data, and the weighted square error of the mark points and motion capture data is minimized.

The law of weighted least squares problem during IK solved by the function:

(1) \begin{eqnarray*}\min _{q}\, \left[ \sum _{i\in markers}{w}_{i}\parallel {{x}_{i}}^{\exp \nolimits }-{x}_{i}(q){\parallel }^{2}+\sum _{j\in unprescribed-coords}{\omega }_{j}({{q}_{j}}^{\exp \nolimits }-{q}_{j})^{2} \right] \end{eqnarray*}

Where, q is the vector of the generalized coordinates solved, *X*_i_^exp^ is the experimental location of mark i, *X*_i_(*q*) is the position of the corresponding mark points in the model (depending on the coordinate value), *q*_*j*_^exp^ is the experimental value of coordinate j, and set their experimental values to the specified coordinates.

**Figure 7:**
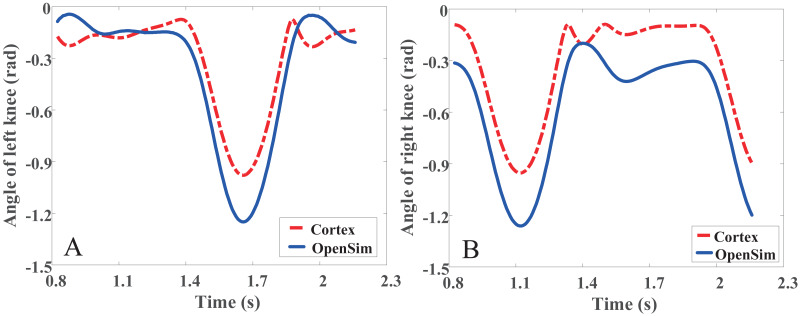
The angle of knee with Cortex & OpenSim. (A) The angle of left knee. (B) The angle of right knee.

The comparison between the knee angle analyzed by Cortex data acquisition software and the knee angle analyzed by OpenSim musculoskeletal simulation software is shown in Fig. 7. The data in the figures represents the gait information of 1.5 cycles. The overall trend of knee joint angle obtained by two methods are similar and can be seen from the figures. However, there are still some differences. It can be seen that the variation range of knee joint angle obtained by using musculoskeletal simulation software OpenSim is larger and the variation trend is more stable. The reason of this phenomenon maybe is the Cortex software get the angles just by simple calculating with the collected position data. OpenSim combines the characteristics of musculoskeletal model in the calculation of joint angles, so the joint angles obtained by OpenSim are better than Cortex.

#### Inverse dynamics (ID)

The ID problem refers to: given the position q, velocity $\dot {q}$ and acceleration *q* of each joint of the robot at a certain moment, calculate the driving force (include: states or motion) imposed on each joint at this time. The ID can be solved by the Newton-Euler equation or the Lagrange equation.

Dynamics is the study of motion, the forces and torques that cause motion. The purpose of ID is to estimate the forces and torques required to produce a particular motion, and the results also used to predict how muscles contribute to motion. In order to calculate the forces and moments, the system’s equations of motion need to be solved iteratively. The motion equations are derived from the motion description and the mass property of the model. In the solution process, the IK is employed to calculate the joint angle and the ground reaction force during the experiment. And combined with the dynamic equilibrium conditions and boundary conditions, the net forces and moments at each joint are obtained.

When solving the ID problem, the data of motion and force measuring platform are needed to ensure that the number of equations of motion more than unknowns (degrees of freedom), which turns the problem into a statically indeterminate problem. The error of experimental motion data and the inaccuracy of the model will lead to Newton’s second law *F*=m*a invalid. In order to solve this dynamic discontinuity problem, residual forces and torques are introduced into a specific section of the model. The following equation is established, which relates the ground reaction moment to the residual moment. Where, ${\overrightarrow {F}}_{\exp }$ is the ground reaction moment and ${\overrightarrow {F}}_{residual}$ is the residual moment.

(2) \begin{eqnarray*}{\overrightarrow {F}}_{\exp \nolimits }+{\overrightarrow {F}}_{residual}=m\cdot \overrightarrow {a}\end{eqnarray*}

Inverse_Dynamics.sto: is generated by inverse kinetic operations, including time series, net joint moments of each bone joint, etc.

**Figure 8:**
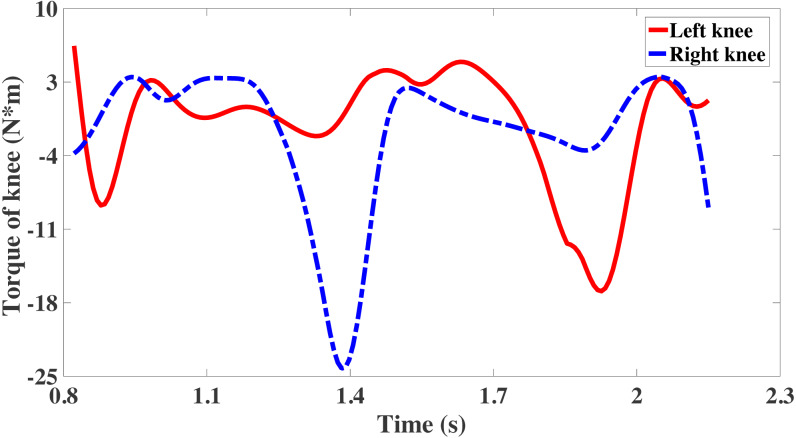
The torque of knee joint from OpenSim.

The net joints acting torque of the hip joints in three motion states are shown in Fig. 8. The net joint acting moment of hip joint is same to the angle of the hip joint, including adduction, flexion and rotation. Torque is drives of the body segments, therefore corresponds to the joint angles of the hip joint. The net joint torque of the knee joint is the joint torque connecting the thigh and the calf, which is significant in the research of exoskeleton rehabilitation robots of lower limbs.

## Control system design

### PD controller

The PD controller is one of the most widely used and effective methods in the field of robotics. As we all know, a PD controller is sufficient to stabilize any kind of rigid manipulator near the reference position. In particular, even when the inertia and friction parameters of the robot are unknown, it can be guaranteed to be asymptotically stable. Under the premise of ignoring friction and other disturbances, Kelly (1993) developed a PD controller with adaptive desired gravity compensation and demonstrated the closed-loop global asymptotically stability that obviates LaSalle’s theorem. In order for the robot to walk like a human, the PD controller is employed to accurately control the robot’s posture and gait, and the stability of the robot is maintained through the feedback system. Putri & Machbub (2018) designed a PD controller with Center of Mass (COM) as system feedback, and verified the effectiveness of PD controller on the Bioloid GP under uneven environments with many obstacles. Hu, Wang & Wu, (2021) proposed a robust adaptive PD-like control based on healthy human gait data. Zhou et al. (2021) proposed a trajectory deformation algorithm and chose a PD position controller to ensure the trajectory tracking effect. Ali et al., (2018) used a fuzzy PID-based position control method in the design of the upper limb rehabilitation robot system to solved the robot’s precise position/force control under the imprecise model. Han, Wang & Tian (2020) used intelligent PD controllers for the motion control of the lower extremity rehabilitation exoskeleton. This method uses a linear state observer to compensate for the control input, solves the inaccuracy of the exoskeleton robot model and the interaction between the human and the exoskeleton. In reality, the robot is a multi-degree-of-freedom, mutually coupled nonlinear system, the performance of robot system depends highly on the availability of high quality differential signal based on the non-continuous measured position signal.

#### Dynamics

Fourier is a rigid body robot, in the absence of friction and other disturbances, the dynamics of a serial n-link rigid robot can be written as:

(3) \begin{eqnarray*}M(q)\ddot{q}\,+D(q,\dot {q}\,)\dot {q}\,+G(q)=\tau +{\tau }_{OpenSim}.\end{eqnarray*}

We simplify Fourier to a 2-link rigid robot, where, *q* is the 2 × 1 vector of joint angle, *τ* is the 2 × 1 vector of joint torques, *τ*_*OpenSim*_ is the 2 × 1 vector of joint torques from the OpenSim software, *M*(*q*) is the 2 × 2 symmetric positive definite manipulator inertia matrix, $D(q,\dot {q})$ is the 2 × 1 coriolis force and centrifugal force matrix, *G*(*q*) is the 2 × 1 gravity matrix. In this paper, *τ*_*OpenSim*_ as a reference torque input to the controller, to realize the anthropomorphic torque output of the exoskeleton robot.

The PD control law is given as flowing:

(4) \begin{eqnarray*}\tau ={K}_{d}\dot {e}\,+{K}_{p}e-{\tau }_{OpenSim}\end{eqnarray*}

where, *K*_*d*_ and *K*_*p*_ are the 2 × 2 symmetric positive definite matrices, *e* = *q*_*d*_ − *q*, *q*_*d*_ is the desired joint angle. The *τ*_*OpenSim*_ is a bounded matrix.

\begin{eqnarray*}{T}_{\mathrm{min}}\leq \left\| {\tau }_{OpenSim} \right\| \leq {T}_{\mathrm{max}}. \end{eqnarray*}

Eqs. (3) and (4) imply.

(5) \begin{eqnarray*}M(q)({\ddot{q}\,}_{d}-\ddot{q}\,)+D(q,\dot {q}\,)({\dot {q}\,}_{d}-\dot {q}\,)+{K}_{d}\dot {e}\,+{K}_{p}e=0.\end{eqnarray*}

Then the $M(q)\ddot{e}+D(q,\dot {q})\dot {e}+{K}_{p}e=-{K}_{d}\dot {e}$ is obtained. Considering the candidate Lyapunov function,

(6) \begin{eqnarray*}V= \frac{1}{2} \dot {{e}^{T}}\,M(q)\dot {e}\,+ \frac{1}{2} \dot {{e}^{T}}\,{K}_{p}\dot {e}\,\end{eqnarray*}

Eq. (6) is position definite, and the time derivative of the function,

(7) \begin{eqnarray*}\dot {V}\,=\dot {{e}^{T}}\,M\ddot{e}\,+ \frac{1}{2} \dot {{e}^{T}}\,\dot {M}\,(q)\dot {e}\,+\dot {{e}^{T}}\,{K}_{p}e.\end{eqnarray*}

There is an oblique symmetry feature: $\dot {M}(q)\text{-2}D(q,\dot {q})=0$. Substituting the condition into Eq. (5), get

(8) \begin{eqnarray*}\dot {V}\,=\dot {{e}^{T}}\,(M\ddot{e}\,+D\dot {e}\,+{K}_{p}e)=-\dot {{e}^{T}}\,{K}_{d}\dot {e}\,\leq 0.\end{eqnarray*}

Then, the stability of design control system can be guaranteed.

#### Results

**Figure 9:**
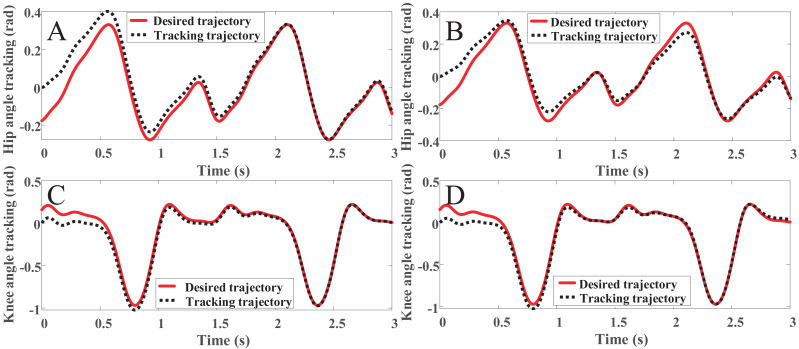
(A) The hip angle track with OpenSim. (B) The hip angle track with link rod model. (C) The knee angle track with OpenSim. (D) The knee angle track with link rod model.

**Figure 10:**
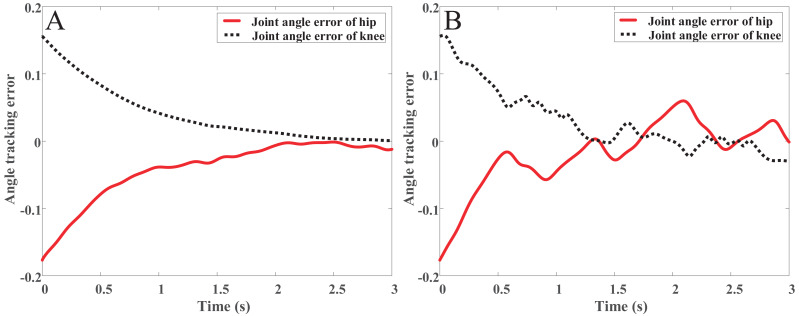
(A) The angle track error with OpenSim. (B) The angle track error with link rod model.

The simulation is carried out in MATLAB-Simulink, and the results are shown in Figs. 9 and 10. Figures 9A, 9C and 10A are the joints angle tracking and tracking error of OpenSim combined with PD controller, respectively. Figures 9B, 9D and 10B are the joints angle tracking and tracking error of the PD controller, respectively. Comparing Figs. 9A and 9B, it can be found that the PD controller combined with OpenSim has a better tracking effect, after the tracking, error-free tracking can be achieved, and the controller that only uses the PD control can clearly found that there is still an error in the peak position of the gait angle in the later stage of the tracking. In the method proposed in this paper, the joint torque from OpenSim plays a good role in compensating for the control of the controller. Comparing Figs. 10A and 10B, we can be found that the PD controller combined with OpenSim has a better tracking effect, the tracking error is smoother with only small fluctuations, and the stability is higher. The patient can enter the rehabilitation training comfortably, and achieve the safety and comfort of the rehabilitation training.

The parameter of PD controller, such as: Kp, Kd. Kp = diag(150, 150), Kd = diag(120, 120). The performance indexes of the two controllers designed are shown in Table 3.

The RMSE, ISE, and ITSE indicated that the two controllers are almost the same, suggesting that they have faster response speed and smaller oscillation. In terms of the properties of IAE and ITAE, the controller with OpenSim output torque is slightly better than the PD controller’s transient response and the transient response oscillation is smaller.

## Conclusions

The collected real human gait data combines with the human musculoskeletal model in this paper, then obtains human motion characteristics by inverse kinematics analysis. These motion characteristics were employed to design the controller and verify it in Simulink. The simulation results show that this method is more flexible and anthropomorphic in the exoskeleton robot control.

**Table 3:** The performance indexes of the two controllers.

	OpenSim with PD controller		PD controller only	
ERROR	Hip error	Knee error	Hip error	Knee error
RMSE	1.7 × 10^−4^	1.6 × 10^−4^	1.4 × 10^−4^	1.5 × 10^−4^
ISE	0.010	0.009	0.008	0.009
ITSE	0.003	0.003	0.004	0.003
IAE	0.1219	0.1164	0.1105	0.1100
ITAE	0.0845	0.0790	0.1076	0.0813

Research innovation points summary shown as follows:

(1) The human biological musculoskeletal dynamics model was identified using OpenSim and the real human gait of the experimental data source. Combining the human lower limb movement characteristics extracted from the gait data with the musculoskeletal model established by OpenSim, the musculoskeletal model is based on the physiological characteristics of the human body (muscle and tendon characteristics, skeletal and tendon connection structure, nervous system, etc.). This method will obtain more accurate gait kinematics and gait dynamics data. Compared with the connecting rod model, this model has a better human-like design, so the precision and accuracy of the model are better than ever.

(2) The humanoid control of robots based on human gait data of normal human gait movements was discussed. The PD controller design and the simulation results based on experimental were analyzed. Experimental results have shown that the control strategy based on OpenSim and PD controller is more in line with the characteristics of human kinematics and physiology. Compared with only using the PD controller, this method has better trajectory tracking effect, faster adjustment time, and more comfortable patient rehabilitation experience.

## Supplemental Information

10.7717/peerj-cs.657/supp-1 Supplemental Information 1 MATLAB simulink code

10.7717/peerj-cs.657/supp-2 Supplemental Information 2 Raw data

## Additional Information and Declarations

### Data Availability

The following information was supplied regarding data availability: The codes and the raw data are available in the Supplemental Files.
